# Seeds and the city: a review of municipal home food gardening programs in Canada in response to the COVID-19 pandemic

**DOI:** 10.1057/s41599-022-01301-6

**Published:** 2022-08-17

**Authors:** Janet Music, Lisa Mullins, Sylvain Charlebois, Charlotte Large, Kydra Mayhew

**Affiliations:** 1 Faculty of Arts and Social Sciences, Dalhousie University, Halifax, NS Canada; 2 Faculty of Management, Dalhousie University, Halifax, NS Canada; 3 Agri-Food Analytics Lab, Dalhousie University, Halifax, NS Canada

**Keywords:** Social policy, Geography

## Abstract

In the wake of the COVID-19 pandemic in Canada, home food gardening articles have saturated popular media outlets. Home food gardening is more popular than ever, and community gardens and community greenhouses are at capacity with long waiting lists for plots. Several local governments across the country are also participating in the food gardening craze. This study compares 19 municipal urban home food gardening programs that ran in 2020. These municipalities provided program participants with free gardening supplies and instructions on how to grow food at home. This study reveals a complicated relationship among municipalities, food gardening programs and household and community food security. The study also determines that the social and emotional challenges brought about by the COVID-19 pandemic are somewhat alleviated through gardening. Ultimately, municipalities are limited in their policy capacities to adequately move the needle on food insecurity in Canada.

## Introduction

Home food gardening has become popular in the wake of the COVID-19 pandemic for many Canadians. In 2020, residential food gardening supplies were completely sold out province-wide in some instances, and retail garden centers reported record profits; 2021 is similarly busy and profitable (Brehaut, 2020; CBC News, 2021; Helmer, 2020; Klinkenberg, 2020; Radio-Canada, 2021; Sharpe, 2021). In a cross-national survey conducted in late September 2020, 51% of Canadians reported that they grew at least one vegetable or fruit at home, with one out of five gardeners new to home food production in 2020 (Mullins et al., 2021). Regardless of city, town, village, or hamlet in Canada, non-commercial, urban food gardening has citizens committed to growing their own vegetables and fruits as far north as the 68th parallel (Brown, 2020; Pigalak, 2020). In the face of the rapid onset of widespread socio-economic upheavals caused by COVID-19, all levels of government sought ways to support the physical, emotional, and mental health of Canadians. Municipal food gardening programs were part of a creative municipal response to the pandemic.

Urban home food gardening enjoyed a prolonged *zeitgeist*-moment in Canada in 2020 and 2021. Canadians sought activities safely undertaken at home. Growing food allows for control over a source of food and guarantees a supply of fresh produce, contingent on the weather. Home food gardeners report that gardening contributes to positive mental and emotional well-being (Chalmin-Pui et al., 2021; Mullins et al., 2021; Theodorou et al., 2021). There is historical precedence for turning to food gardening in times of crisis. In 2020, Canadian citizens and local governments have appropriated the discourse and emotional ties of war Victory Gardens in the “fight” against COVID-19 (Music et al., 2021). City staff in North Battleford, Saskatchewan (pop. 14,315) planted a Victory Garden on municipal land and donated the harvests to food banks and community food centers (City of North Battleford, 2020). In Winnipeg, Manitoba (pop. 705,244), a Victory Garden on city property was proposed and spearheaded by a city councillor: the city and a local non-profit partnered to plant and tend to their Victory Garden, with the harvest donated to low-income residents via a food box program (Winnipeg Food Council, 2020).

Academic literature in this area focuses on gardeners, as well as community gardens. Community gardens increase food security and food literacy (Algert et al., 2016; Cochran and Minaker, 2020; Garcia et al., 2018; Gregory et al., 2016; Lowan-Trudeau et al., 2020), are sites of community connectivity and cohesion (Kingsley et al., 2020; Lucas and Li, 2020; Reese, 2018), and benefit physical and mental health (Alaimo et al., 2016; Al-Delaimy and Webb, 2017; Genter et al., 2015; Hartwig and Mason, 2016; Tharrey et al., 2020). Community gardens and other urban agricultural gardening initiatives are proven to increase neighborhood property values (Cochran and Minaker, 2020; Rosan and Pearsall, 2017). Community gardens and urban agriculture are increasingly seen as a key component of urban planning, a marked change from just a decade ago (Cabannes and Marocchino, 2018; Kroeker, 2017; Rosan and Pearsall, 2017; Smith et al., 2021; Soderholm, 2015).

Home food gardening has not received the same level of academic attention, especially in Canada, though the COVID-19 pandemic is encouraging more scholars to investigate domestic food production. Home food gardens are difficult to study, as identifying home food gardeners relies on popular self-identification and/or complex, time-consuming processes; home food gardening studies can also be complicated by the many different forms such a garden can take (Mullins et al., 2021; Smith et al., 2013; Taylor and Lovell, 2014, 2015). In Canada, more individuals grow food at home than in community gardens (Duchemin, 2020; Duchemin and McClintock, 2020; Mullins et al., 2021). Lal (2020) provides an overview of the benefits and possibility of home food gardening and urban agriculture in light of the COVID-19 pandemic, but his global perspective is, by definition, too broad to inform specific policies and programs. Small studies on North American food gardening have been conducted in Guelph (CoDyre et al., 2015), Toronto (Kortright and Wakefield, 2011), and Montréal, Canada (Duchemin and McClintock, 2020), and in San Jose, USA (Gray et al., 2014). These studies were conducted prior to the onset of the pandemic and its associated socio-economic upheavals. This calls into question the relevance of their conclusions around the frequency of home food gardening, but their findings regarding habits and motivations of gardeners are still valid (Mullins et al., 2021).

**Table 1:** Municipalities with home food gardening programs in 2020.

Municipality	Province	Population^a^	Average household income^b^	Prevalence of low- income persons (%)^c^	Unemployment rate (%)^d^	In rented housing (%)^e^	Urban/rural	Plant hardiness^f^	Latitude
Montréal^g^	Québec	1,704,694	$69,047	22.7	9.3	63.3	Urban	6a	45.5017°N
Ottawa	Ontario	934,243	$106,372	12.6	7.2	34.3	Urban core, 80% of land rural	5a/5b	45.4215°N
Brampton	Ontario	593,638	$98,855	11.3	8.3	20.0	Urban	6a	43.7315°N
Surrey	British Columbia	517,887	$93,586	14.8	6.5	28.9	Urban, with significant rural areas	8a-8b	49.1913°N
Halifax Regional Municipality (HRM)	Nova Scotia	403,131	$86,778	14.8	7.3	39.9	Urban core, 80% of land rural	6b	44.6488°N
Greater Sudbury	Ontario	161,531	$90,179	12.8	8.3	34.2	Urban core, majority of land rural	4b	46.4917°N
Ajax	Ontario	119,677	$112,569	9.4	8.2	13.8	Urban	6a	43.8509°N
Waterloo	Ontario	104,986	$108,411	13.7	6.9	31.2	Urban	6a	43.4643°N
Red Deer	Alberta	100,418	$110,394	10.0	10.2	34.3	Urban, with some rural land	3b-4a	52.2690°N
Victoria	British Columbia	85,792	$69,383	19.8	6.0	60.6	Urban	8b-9a	48.4284°N
Grande Prairie	Alberta	63,166	$118,775	7.2	9.7	34.7	Urban core, with significant rural areas	3b	55.1707°N
Saint-Eustache	Québec	44,008	$79,560	10.1	6.8	30.2	Urban and rural (50/50)	5b	45.5618^o^N
Brant	Ontario	36,707	$105,113	7.2	4.3	13.3	Rural, with 1 urban center	6a	43.1527°N
Chambly	Québec	29,120	$97,556	6.5	4.0	23.6	Urban and rural (50/50)	5b	45.4618°N
Saint-Constant	Québec	27,359	$94,356	5.5	4.8	18.4	Urban core, 70% of land rural	6a	45.3699°N
Deux-Montagnes	Québec	17,496	$83,297	10.2	6.1	28.0	Urban	5b	45.5333°N
Sainte-Anne-des-Plaines	Québec	14,421	$78,784	10.1	6.1	25.1	Rural, with 1 urban center	5b	45.7666°N
Lorraine	Québec	9352	$188,705	4.8	4.5	3.8	Urban	5b	45.6594°N
L'Épiphanie^h^	Québec	5493	$70,496	16.6	5.2	39.0	Rural, with 1 urban center	5b	45.8508°N

^a, b, d, e^Statistics Canada, 2016 Census (Statistics Canada, 2019).

^c^Statistics Canada, 2016 Census. Calculated based on after-tax income; the low-income threshold for an individual was $24,021 and $48,023 for a family of 2 adults and 2 children under 15 years of age Statistics Canada, 2019.

^f^Plant hardiness indicates a particular plant species’ ability to grow in a geographic location. In Canada, the scale ranges from 0 to 9a, with each number having an “a” and “b”, where 0a = no plant can grow outside without constant, continual human intervention. Natural Resources Canada calculates plant hardiness zones using several factors, including amount of rainfall from June to November, monthly mean of daily maximum and minimum temperatures, and how many frost-free periods there are in a given period. Many seed packets include the range of plant hardiness zones they are suited for on their packaging.

^g^Montréal has a complex municipal structure. This table contains data about the Ville de Montréal only, that is, the legal entity with that name. See the Results section for more information.

^h^The municipality of L'Épiphanie and the neighboring Parish of L'Épiphanie merged in 2018, becoming la Ville de L'Épiphanie (pop. est. 8900 in 2020). 2016 Census data reflects the old municipality of L'Épiphanie only.

This study offers an evaluation of Canadian municipal gardening programs (see Table 1) that support residents in home food production and assesses the extent these achieve program success in key food policy areas by highlighting four notable examples. These municipalities provided program participants with free gardening supplies and instructions on how to grow food at home. Food gardening programs potentially make meaningful contributions to household and community food security. Municipalities, in keeping with specific provincially mandated policies, can support programs that alleviate the social challenges brought on by the COVID-19 pandemic.

## Methodology

The present study of municipal home food gardening programs in the wake of COVID-19 came from a data set of urban agricultural policies and programs for 702 municipalities. Inclusion requires a municipality to have at least one urban center with a population of at least 2000 people. Our definition of urban was influenced by Statistics Canada’s concept of population center: “A population center (POPCTR) has a population of at least 1000 and a population density of 400 persons or more per square kilometer, based on the current census… All areas outside population centers are classified as rural areas”. To have a manageable number of data points, we raised the minimum population threshold but did not adhere strictly to a population density of 400 persons per square kilometer. Population, population density, and the land/territory area of each municipality are taken from the Government of Canada’s 2016 Census (Statistics Canada, 2019). It should be noted that the 2021 Census is in progress at the time of writing this article, with various datasets scheduled to be released starting in February 2022; it is unlikely that a significant number of small municipalities will have crossed the 2000-person population threshold to qualify for inclusion in this study. Given the physical size of Canada and its varied geographies, the population distribution of Canada’s four smallest provinces and the three territories dictated our population criteria of 2000+ residents.

Qualifying municipalities were identified using official provincial and territorial websites, satellite imagery from Google Maps, and the Statistics Canada website. The provincial/territorial ministry responsible for municipalities maintains a list of all municipalities and their population, taken from Statistics Canada’s most recent census. Each municipality was then checked on Google Maps to see if it had an urban center. If so, the name of the urban center was searched in the census data to determine its population, as, among other layers, the census provides data for all legally incorporated municipalities and for population centers within those municipalities.

Municipal home food gardening programs were discovered using various search terms (see Table 2) on official municipal websites and across the internet using Google. Google was used to locate news and newspaper articles and videos about the programs, to supplement official municipal press releases and, in some cases, provide follow-up at the program’s conclusion. Searches in Google were conducted in English or French, depending on the province.

**Table 2:** Search terms used to discover municipal home food gardening programs.

English	French
Home food garden	Potager domestique
Growing food	Potager/Jardiner
Backyard garden	
Free seeds and soil	Semences/grains et terreau gratuit/libre
Urban agriculture	Agriculture urbaine

To identify and evaluate municipal home food gardening programs, and more general urban agriculture policies and programs, this study used publicly available municipal documents such as reports, official policies, and bylaws from official government websites, as well as articles and media from trusted news outlets, with local newspapers (online editions) as key information resources. Owing to the severity of the COVID-19 pandemic in various Canadian municipalities throughout the first half of 2021 and the country-wide scope of this research, only sources of information available online were consulted.

A municipality was considered to have a home food gardening program in response to COVID-19 if the program originated in or was modified in 2020. Municipalities offering free garden compost only were excluded, as many Canadian municipalities have been doing so for more than a decade. The municipality must have provided funding for the program by direct cash contribution or in-kind services and/or have played some role in the administration or delivery of the program, whether it was a behind-the-scenes coordination role, or direct-service delivery to participants. Programs that were delivered by public library systems were excluded from this study. Though municipalities fund public libraries, library systems act independently—municipalities rarely dictate their programming and service delivery beyond broad areas. Participation in the home food gardening program must have been limited to residents of their respective municipalities. Programs had to involve at least one transfer of physical material, such as the distribution of free vegetable seeds to participants. While many more than 20 municipalities offer gardening information resources online, and many of these were updated or added in 2020, these municipal offerings were excluded from this study. It is possible there were other municipal home food gardening programs in Canada running in 2020 that were similar to these 20. In smaller municipalities, it is possible that news stories and announcements from 2020 were removed from their websites. In very small municipalities, it is also possible that information about food gardening programs was circulated through print-based community newsletters or by word of mouth only.

Four municipalities identified as having a robust home gardening program in 2020 as a response to the COVID-19 pandemic were contacted. Unstructured interviews were conducted between May and July 2021 with staff from the Halifax Regional Municipality, as well as staff from Cultiver Montréal, and with a municipal councillor from the City of Brampton. Each interview took approximately one hour and was conducted via videoconferencing. Email interviews were conducted with staff from the City of Victoria. Transcripts from this interview totaled four pages, single spaced. The municipalities of Victoria, Brampton, Halifax, and Cultiver Montréal shared internal documents with the authors, adding to the availability and precision of information regarding their home food gardening programs. All videoconference interviews were transcribed using Microsoft Teams. Telephone conference transcription was conducted in Microsoft Word.

The transcripts of the interviews, as well as the policy documents, were coded into themes, which emerged through the codes developed from a grounded perspective. Key themes were identified and grouped by scanning descriptions of programs and desired program outcomes that were universal among the texts. The characteristics of each program-case were examined, and these cases were compared to look for patterns between offerings based on program delivery and outcomes as well as municipality size, urban/rural context, and province.

## Results

Of 702 Canadian municipalities, only 19 (2.7%) were found to have a municipality-based active home food gardening program in 2020 (Table 3); this dropped to 1.7% in 2021. The number of municipal home food gardening programs dropped to 12 in 2021.

**Table 3:** Overview of municipal home food gardening programs in 2020.

Municipality	Program name	Number of participating households^a^	Targeted to low-income residents	Seeds	Seedlings	Soil	Container(s)	Partner organization(s)	Program ran in 2021^b^
Montréal	Festival Cultiver Montréal	Unknown	No	Yes	Yes	Yes	Yes	Cultiver Montréal	Yes
Ottawa	Seeds and Soil Home Garden Project	Just under 3000	Yes^c^	Yes	Yes (some)	Yes	No	Just Food (primary); several other partner organizations	No
Brampton	Backyard Garden Program	6000	No	Yes	Yes (some)	Yes	No	–	Yes
Surrey	Yard to Garden Program	60	No	Yes	No	Yes	No	Seeds of Change (primary); United Way	No
Halifax Regional Municipality (HRM)	Food Gardening @ Home	1. 350+ 2. 260+ 3. 80+	Yes	Yes	No	Yes	Yes	4 partner organizations	No
Greater Sudbury	Home Garden Project	1. 288 2. 78 3. Unknown	No	Yes	No	Yes	Yes (some)	Sudbury CGN (primary); several other partner organizations, local businesses	No
Ajax	Small Garden Kit Giveaway	500	No	Yes	No	Yes	Yes	2 Partner businesses	No
Waterloo	–	100	No	Yes	No	Yes	Yes	–	No
Red Deer	Home Grown	460+	No	Yes	No	Yes	Yes	–	No
Victoria	Get Growing, Victoria!	5537	No^d^	No	Yes	Yes	No	44 partner organizations	Yes
Grande Prairie	GP Grows	1. 800 2. 700	No	Yes	No	Yes	Yes	–	Yes
Saint-Eustache	Journée Thématique en Environnement	Unknown	No	No	Yes	No	No	–	Yes
Brant	Host Garden Program	20	No	Yes	Yes	No	No	Equal Ground Community Garden	No
Chambly	–	250	No	No	Yes	No	Yes	–	No
Saint-Constant	Mon Petit Jardin Bio	1. 750 2. 500	No	Yes	Yes	No	No	–	Yes
Deux-Montagnes	La Journée Horticole	1800^e^	No	No	Yes	No	No	–	Yes
Sainte-Anne-des- Plaines	La Journée Annuelle de l’Environnement	Unknown	No	No	Yes	Yes	No	3 local businesses	Yes
Lorraine	Journée Verte	400	No	No	Yes	No	Yes	–	Yes
L'Épiphanie	Journée de l’Environnement	525	No	Yes	Yes	Yes	No	–	Yes

^a^Some municipalities had more than one cohort of the same program, or two or more programs running, under the same program title. Grande Prairie’s numbers are approximate.

^b^With the exception of the City of Victoria, all home food gardening programs were modified somewhat for their 2021 iteration.

^c^Low-income households were given priority; however, registration then opened up to all residents.

^d^Some residents were prioritized for Victoria’s Get Growing program: those who were facing food security barriers caused by historical marginalization, those who lost their jobs due to COVID-19, and residents who were immunocompromised (but not necessarily low-income or otherwise marginalized).

^e^This number includes participants who just received flower plants rather than flower and edible herb plants.

### Ville de Montréal—limited offerings

This home food gardening program was delivered by the non-profit urban agriculture group Cultiver Montréal as part of its annual Festival Cultiver Montréal. The municipality provided $45,000 to fund three food gardening initiatives, one of which was the distribution of free seeds and seedlings to residents of Montréal to grow food at home during the COVID-19 pandemic (Olson, 2020; Ville de Montréal, 2020). Montrealers were also able to buy seeds, seedling plants, and other gardening material at greatly subsidized prices from local growers and gardening centers. Owing to pandemic restrictions, Cutliver Montréal organized delivery of all gardening materials to community gardens, other non-profit food organizations, and citizens. It is unclear how many households the program reached, but more than 2800 seedlings were distributed to households and community gardens across the city (Cultiver Montréal, 2020).

It should be noted that the Island of Montréal, with an area of 472.55 km^2^, consists of 16 formal (legal corporations) municipalities; Ville de Montréal is the largest, both in terms of population and land area. Cultiver Montréal runs programs that are island-wide: part of this grant was used for virtual urban agricultural programming, which, given its online delivery, was open to residents island-wide (Cultiver Montréal, 2021; Ville de Montréal, 2020). However, Montréal’s city council specified that free seeds and seedlings were for Ville de Montréal residents and community gardens only, with distribution in ten of the city’s 19 *arrondissements* (Ville de Montréal, 2020). The authors were unable to determine how households were selected to receive free vegetable seeds and seedlings.

### City of Brampton—superseding pandemic programming

Brampton’s Backyard Garden Program was the largest home food gardening program in response to COVID-19. Announced on 14 April 2020, via the city’s website, social media channels, and several local media outlets, the program was the idea of Councillor Doug Whillans, an avid home food gardener and advocate for urban agriculture in Brampton. Interested residents were asked to email the city and, much to Whillans’ surprise, over 3000 residents responded in 24 h. In less than a week, 14,000 Bramptonians had signed up. City staff capped participation at 6000 households, and participants were chosen on a first-come, first-serve basis by city ward to ensure an equal distribution of participants across the municipality. Each garden kit contained seed packets and soil, with some also containing seedlings, and instructions. Participant households could select the amount of soil they wanted and, based on that, were allocated seeds: 765 l of soil (cubic yard) and three packages of assorted vegetable seeds; 380 l of soil and two seed packages; or around 100 l of soil and one seed package (City of Brampton, 2021a, 2021b; Whillans, pers. comm., 2021).

Gardening kits were delivered to each participant. Seeds were sorted, packaged, and delivered to participants by city staff and more than 45 volunteers. Soil, which was bought by the City of Brampton from local gardening and landscaping centers, was delivered to various city facilities; city staff divided up the soil and, with the help of the city fire department, delivered the enormous amount of soil to all the participants. Gardeners who requested 100 l of soil had bags of soil delivered, while all other participants received loose soil. Backyard Garden Program gardeners were asked explicitly to share their harvest with those in need: gardening kits contained a food bank donation form, which had a unique (anonymized) participant number and the locations and hours of some of the city’s food banks. When participants dropped off a produce donation, the food bank used the form to track donations from the program. All participants had received their gardening kits by the end of May 2020 (City of Brampton, 2021b; Whillans, pers. comm., 2021).

### Halifax Regional Municipality (HRM)—limited success

The Growing Food @ Home program was conceived by municipal staff who were members of the municipality’s food policy council. There were three different sets of free gardening material, each aimed at a different group of residents: seed packets, gardening kits, and container gardening kits. Seed packets contained two types of vegetable seeds that could be harvested as microgreens or full-sized vegetables, and were suitable for growing in the ground or in pots; these kits were for HRM residents who had space and soil for gardening at home. Gardening packs had 16 Jiffy pots (biodegradable containers for seedlings), one nine-liter bag of soil, and two seed packets. Gardening kits were for residents who had the space to plant in a garden bed or in an in-ground garden, with the plants started inside and then transplanted outdoors when the weather allowed. Finally, the container gardens were for residents who did not have outdoor land for gardening; they consisted of a seven-gallon container, 30 l of soil, and two seed packets. The kits all included instructions and information on the municipality’s food action plan (Halifax Regional Municipality, 2021a).

The Growing Food @ Home gardening kits was distributed by five partner organizations. The program was targeted at residents of HRM who were experiencing food insecurity in the early months of the COVID-19 pandemic. Instead of holding specific distribution events, the gardening kits were distributed to HRM residents when they picked up or received a delivery of emergency food aid. Staff and volunteers asked residents if they were interested in home food gardening; if the response was yes, they got the best-suited gardening kit to their housing situation while supplies lasted at each specific location. The program was not widely advertised, as it was executed very quickly by city staff and partner organizations (HRM staff member, pers. comm., 2021).

### City of Victoria—aided by climate

In 2020, Get Growing, Victoria!, the city’s pandemic-response home gardening program, had 5537 participant households. Over the course of several months, over 81,500 edible plants, 202 cubic yards of gardening leaf mulch, compost, and wood chips, and gardening instructions were distributed to participants across the city. All the seedlings were grown in city greenhouses and plant nurseries by the city’s parks staff; perhaps not surprisingly, the total number of seedlings grown surpassed project estimates. Get Growing, Victoria! was coordinated and delivered by the City of Victoria, with support from the Urban Food Table (Victoria’s food policy council), local farmers, and the school district, with 44 partner organizations assisting with the physical delivery of plants and soil to residents. The Urban Food Table suggested the idea of a home food gardening program at the very beginning of the pandemic. Participants were not required to register with the municipality, but some distribution sites required advanced registration due to COVID-19 public health measures (City of Victoria, 2021, 2021b).

Climate conditions played a significant part in the city’s efforts to get so many residents gardening and ability to supply vulnerable residents with home-grown fresh produce. Because of Victoria’s climate, the municipality was able to offer two cohorts of Get Growing, Victoria! Soil was distributed in April/May, and seedlings in May/June and August/September. The long growing season enabled more residents to participate as interest in the program spread through neighborhoods. Gardeners had the opportunity to have a double harvest garden, with produce ready for consumption as early as August, and as late as November (City of Victoria staff member, pers. comm., 2021).

Partner organizations and the Victoria school district distributed seedlings and soil at 30 distribution day events held at community centers, recreation centers, parks, and schools; they also delivered garden materials to some residents who were immunocompromised or house-bound for other reasons. Other partner organizations distributed seedlings to participants in their existing programs, including community gardens, while some organizations helped participants care for their garden. Finally, some partner organizations received seedlings to plant in their community gardens, with the harvest going to people needing emergency food access (City of Victoria staff member, pers. comm., 2021).

## Discussion

### Food security and food production

Twelve of the 19 municipalities (63%) cited increased food security as a reason for running their home gardening programs. Of these, only one—Halifax—limited participation to households experiencing food insecurity, while one other—Victoria—publicly declared its goal was to help households experiencing food insecurity and/or restricted access to fresh food. At least 74% of participants in Get Growing, Victoria! self-identified as being disproportionately negatively impacted by the pandemic (City of Victoria staff member, pers. comm., 2021). These home food gardening programs produced significant quantities of food for participants and their communities. Based on a 50% success rate, the City of Victoria calculated that 48,690 kg of produce was grown by Get Growing, Victoria! participants (City of Victoria staff member, pers. comm., 2021). This is a very conservative success rate, considering Victoria’s climate and the fact they distributed healthy seedlings. There are not enough known variables to enable calculation of average harvest yields for each participant in each of the home gardening programs; at a minimum, average garden size in m^2^ plus soil depth is needed (CoDyre et al., 2015; Duchemin and McClintock, 2020). It is also not possible to calculate the cost difference between home-grown and store-bought produce.

The volume of fresh vegetables and fruits that program participants grew improved food security in terms of access to healthy produce. 81.6% of Get Growing, Victoria! participants agreed they had increased access to fresh produce, while 72.7% agreed they consumed more fresh food because of their home garden. Encouragingly, 69.3% of program participants in Victoria said that their participation in the program saved them money on groceries (City of Victoria staff member, pers. comm., 2021). It is—apart from Victoria and Halifax—less sure if program participants were experiencing food insecurity prior to or during the 2020 growing season. The economic demographics of their citizens does not seem to have influenced municipalities in the design and motivation behind their home food gardening programs, again except for Victoria and Halifax (see Table 1). Although many municipalities cited food security, only two of their gardening programs engaged explicitly with those experiencing or at risk for food insecurity. Halifax did not advertise their Food Gardening @ Home program at all; program participants found out about it as they picked up emergency food supplies. Studies show that only 20.6% of food-insecure households will visit a food bank, as many individuals believe food banks take away their dignity and dehumanize them (FoodARC, 2021; Godrich et al., 2019; PROOF, 2019). Had the municipality advertised its food gardening kits, they would have reached more households experiencing food insecurity.

However, Brampton asked program participants to donate surplus harvest to neighbors and community food banks and both Montréal and Victoria donated seeds and seedlings to community gardens to support food bank gardening plots. Brampton’s home food gardening program launched before the province of Ontario decreed that community gardens could open during COVID-19. A significant motivation for its Backyard Garden Program was the question of supply for their food banks: most of Brampton’s many community gardens have dedicated growing space for food banks and community organizations that provide meals to low-income and vulnerable residents (Whillans, pers. comm. 2021). Participants in Brampton’s Backyard Garden Program donated close to 5000 kg of fresh produce to local food banks (City of Brampton, 2021a; Whillans, pers. comm., 2021). Clearly, these home food gardening programs helped households in their communities experiencing food insecurity.

### Creating community connections

Seven of 19 (37%) municipalities explicitly cited community cohesion as a motivation and goal of their home food gardening program, though the programs of several other municipalities fostered community and neighborhood connections. A home food garden affords less opportunity for collaborative gardening, making new friends, and building relationships with community members, but home food gardens are still places of community connections, with (geographically) close neighbors. They are also sites “of civic engagement…where people reflect about the food system and their place in it” (Gray et al., 2014, p. 189); home gardens can inspire community creation and development (Gray et al., 2014). Community connections include those between the municipality and its residents, the municipality and non-profit organizations and neighborhood groups, and between residents of the municipality.

Get Growing, Victoria! did not highlight community connections as a program goal. However, given the number of community organizations involved in its delivery, the program certainly fostered connections between non-profit organizations and the municipality and between non-profit organizations. This collaboration will hopefully lead to partnerships strengthening community food security across Victoria. Participants in the program agreed that their home food gardens helped foster community relationships: 57% felt more connected to other residents of Victoria (City of Victoria staff member, pers. comm., 2021). Activities that fostered these feelings of community togetherness included online gardening groups, work parties, and mentorships, with neighbors and community organizations’ staff and volunteers (City of Victoria staff member, pers. comm., 2021).

### Partnership

Related to creating community connections and fostering inclusivity is the question of municipal partnerships with outside community organizations. The variety of partner organizations involved in the execution of these municipal home gardening programs demonstrates that municipalities have many local resources to support them in their efforts to improve community food security with food gardening initiatives. Interestingly, the two municipalities with the largest programs—Brampton and Victoria—had the least and the most partner organizations, respectively. Partnering with a community organization certainly saves money, in terms of municipal staff work hours. Though non-profit organizations (usually) have precarious financial situations, they have groups of passionate volunteers alongside their paid staff. Also, Montréal is arguably too large to handle the minutia of a free seeds and soil program.

Arguably, Halifax and Victoria were forced to partner with community organizations because of their focus on specific population groups coupled with the absence of pre-registration. The non-profit and grassroots community groups that partnered with the City of Victoria were not all devoted to food security; rather, the city worked with organizations that had proven capacity to run distribution days throughout the city and/or organizations that had established, trusting relationships with vulnerable groups of residents. Two of HRM’s four distribution partners were trusted emergency food providers with longstanding relationships in the communities they serve: Feed Nova Scotia, the umbrella organization for hundreds of food banks across the province and the North End Parent Resource Center, an organization that offers programs and support for families in an historically low-income and racialized neighborhood. One of their other partners is a municipal agency, the Community Mobilization Team (CMT), which exists to support a community after a traumatic incident, and is made up of municipal staff, local residents, and community organizations (Halifax Regional Municipality, 2019). Their fourth partner organization—Halifax Public Library (HPL)—is not a traditional provider of emergency food aid, but HPL shifted their usual food support programs to take-away food distribution early in the pandemic; their supports include healthy snack packs and full lunches, distributed from library branches in neighborhoods with high instances of household food insecurity (HPL staff member, pres. comm., March 2021). Both municipalities had to rely on their many partner organizations to ensure the gardening kits reached their target participants.

### Potential barriers to participation

Each of these 19 home food gardening programs has flaws, especially in terms of social inclusion. Some of these exclusionary tendencies were likely the result of the speed with which the programs were developed and delivered, though others were more likely the result of unconscious bias and systematic exclusion on the part of municipalities and organizers. Very few of these exclusionary tendencies were resolved in the 2021 iteration of these home food gardening programs.

The digital divide is the primary exclusionary feature of all 19 of these municipal home food gardening programs. There exists a digital divide in Canada between urban and rural regions, and within these regions among households with divergent financial means. The COVID-19 pandemic and the switch to online and virtual services across all sectors of society have highlighted the need to ensure all households have an internet-enabled device, with reliable, affordable high-speed internet access, on a stable and affordable electrical grid (Lai and Widmar, 2021; Reddick et al., 2020; Tiku, 2021). All 19 home food gardening programs were first announced online and the majority (13) required online registration, though L'Épiphanie also offered telephone registration. Internet and digital information literacy skills were required for registration in many of these municipal gardening programs, which presumes a level of digital literacy that can by no means be assumed to exist. For future iterations of their gardening programs, municipalities need to keep the digital divide at the forefront of their planning for home food gardening programs to ensure that all residents have an opportunity to participate; for example, a municipality could set aside a certain number of program spots for participants to register with the help of the public library or non-profit community group.

Many home food gardening programs provided instructions and supporting online resources only in English or French. With one exception, municipalities did not account for individuals with low literacy skills or adults with little English/French language skills; this is especially disappointing for municipalities with significant populations of newcomers to Canada.

Halifax made sure its instructions for the Food Gardening @ Home kits were heavily illustrated and written using simple sentences and vocabulary to promote the inclusion of those individuals with low English literacy or limited English language skills. Language and literacy barriers are a relatively simple exclusionary tendency to fix in terms of home food gardening programs: it is a matter of translation. Municipalities and partner community organizations should, like Halifax, have image-based instructions.

Brampton’s program is geared towards those who own a detached or semi-detached single-family home, based on the types of seeds and seedlings distributed or the program’s official description. This is problematic for programs that emphasized the link between home food gardening and food security: low-income individuals are more likely to live in rented housing and/or in multi-unit buildings with limited personal outdoor space. For households that live in rental housing, landlords may have restrictions on outside landscaping or already have the property’s outdoor space landscaped in such a way that makes a food garden impossible. More and more adults are choosing to live in apartments or condominiums, especially in urban centers like Montréal; the authors’ research showed that 19% of food gardeners grew at least some food on balconies (Mullins et al., 2021). Also, many older individuals live in apartments or condominiums, and are more prone to mobility issues, making a container garden the best option for growing their own food.

The selection of crops for all 19 municipal home food gardening programs is based on conventional White, Western European food preferences. In highly multicultural municipalities, at least one-quarter of seeds and seedlings on offer should speak to other prominent national or cultural food traditions. There are international non-invasive crops that grow well in Canadian climates, as many community gardens and household gardens have proved (Lucas and Li, 2020; Toughill, 2018). Food is a vital component of culture: for many individuals in Canada, getting access to culture-specific vegetables and fruits is impossible, either due to cost or the simple fact that they do not exist in Canada (Toughill, 2018). Of course, not every culture can be represented, but sizeable national/cultural populations of a municipality could. More culturally diverse vegetables and fruits are especially important for The City of Brampton: 52% of the population are immigrants, with 40% coming from India (Statistics Canada, 2019). Including seeds or seedlings of ridged gourd or purple yam, or a herb selection commonly used to make curry, alongside common “Canadian” vegetables like cucumber and carrots, would perhaps make the Backyard Garden Program relevant to more residents. It would also present an opportunity for program participants to grow new foods from different cultures.

Offering traditional Indigenous seeds and seedlings to home food gardening program participants represents an educational opportunity for non-Indigenous individuals, as well as those who identify as Indigenous, especially younger generations. It would also present an opportunity for municipalities to partner with Indigenous community organizations active in their regions.

### Food gardening policies

The creation of a home food gardening program is a municipality actualizing its policy statements. Fifteen of the 19 municipalities have at least one high-level policy document in which urban food gardening is identified as beneficial to the municipality, whether it be for food security, environmental sustainability, or recreational reasons (see Table 4). All these discussions about food gardening involve community gardens, while home food gardening, edible landscaping, and other urban agriculture initiatives are occasionally discussed. Municipal policy priorities are extremely important because they determine which initiatives get funded. To determine policy regarding food gardening, the official municipal plan, environmental sustainability plan, parks master plan, and urban agriculture plan were considered in each of the 19 municipalities with home food gardening programs in 2020.

**Table 4:** Food gardening in significant municipal plans and reports, and food gardening programs.

Municipality	Food gardening in official municipal plan	Food gardening in environmental and/or sustainability plan	Food gardening in parks/green spaces master plan	Specific urban agriculture plan/policy	Edible landscaping project(s) and/or food forests on municipal land	Community garden(s) on municipal land, run by municipality	Community garden(s) on municipal land, run by community organization(s)
Montréal	Yes	Yes	No and Yes^a^	No and Yes^a^	Yes	Yes	Yes
Ottawa	Yes	Yes	No	No	No	Yes	Yes
Brampton	Yes	Yes	Yes	No	No	Yes	Yes
Surrey	Yes	Yes	Yes	No	Yes^b^	No	Yes
Halifax Regional Municipality (HRM)	Yes	No	No	No	No	No	Yes
Greater Sudbury	Yes	Yes	Yes	No	Yes^b^	No	Yes
Ajax	Yes	Yes	No	No	No	No	Yes
Waterloo	Yes	Yes	No	No	No	No	Yes
Red Deer	Yes	Yes	No	No	Yes	Yes	Yes
Victoria	Yes	No	Yes	No	Yes^b^	No	Yes
Grande Prairie	No	No	Yes	No	Yes	No	No
Saint-Eustache	No	Yes	No	No	No	No	Yes
Brant	No^c^	No	Yes	No	No	No	Yes
Chambly	Yes	No	No	No	No	No	Yes
Saint-Constant	No	Yes	No	No	Yes	Yes	No
Deux-Montagnes	No	No	No	No	No	No	Yes
Sainte-Anne-des- Plaines	No	No	No	No	No	Yes	No
Lorraine	Unknown^d^	No	No	No	No	No	No
L'Épiphanie	No	No	No	No	No	No	No

^a^Some *arrondissements* in Montréal mention food gardening in these plans, others do not. La Ville de Montréal does not have an overarching parks master plan or an urban agriculture plan/policy.

^b^Edible landscaping initiatives and/or food forests in these municipalities are managed by community organizations; the municipalities provide some funding or in-kind support, in addition to the land.

^c^At the time of writing (July 2021), the County of Brant is nearing completion of its new Official Municipal Plan.

^d^Not available online.

An official municipal plan involves land use and service planning; it is a goal-setting document for the development of the total municipality, which municipal councillors and staff refer to for decision-making and the creation of all initiatives. Plans are updated and completely re-written at set intervals, dependent on provincial legislation, with most municipalities updating plans every 3–5 years, and creating new plans every 10–30 years. Eleven of the 19 municipalities have some discussion of food gardening in their official municipal plan, which legitimates their home food gardening programs from a policy perspective. Most of these are broad statements supporting urban agriculture and local food production for reasons of community food security, health, and environmental sustainability.

Nine of the 19 (45%) municipalities identify environmental sustainability as a motivation for their home food gardening program. Food gardening is present in half (10 of 19) of the municipalities’ environmental sustainability plans or green/environment policies, yet these two do not coincide in all cases. Brampton cited environmental sustainability as a justification for their home food gardening program, which reflected food gardening objectives present in their environmental master plans (City of Brampton, 2020).

Montréal is the only municipality with a home food gardening program to have an urban agriculture plan or policy—even then, only some *arrondissements* have them, and their plans are not applicable to the entire city. Other municipalities discuss urban agriculture in their official municipal plans. Halifax has food policy documents that include sections on food gardening as part of larger discussions of urban agriculture and, as previously mentioned, three municipalities include discussions of food gardening and urban agriculture in their environmental sustainability plans. A separate, specific policy and/or plan for urban agriculture indicates that food gardening is a significant priority for the municipality, not just part of a larger priority. There is only a handful of Canadian municipalities with urban agriculture plans/policies, with almost all of them created in the past four years. However, there are signs this is changing: in 2020 and 2021, close to a dozen municipalities in Quebec released official urban agriculture plans. Perhaps not surprisingly, many of these municipalities cite the COVID-19 pandemic as a reason why local urban food production is important, though the genesis of many of these plans was pre-pandemic.

There is no correlation between strong community gardening programs and the existence of a home food garden program. Many of the 683 municipalities without a home food gardening program in 2020 had robust community garden programs and edible landscaping initiatives. Montréal has hundreds of community gardens, many of which are on municipal land but run by a neighborhood group or non-profit organization, while some are run directly by the city. The city also has a strong tradition of home food gardening and urban agriculture more generally (Duchemin, 2020), which makes it somewhat surprising that the municipality was not more involved in its home food gardening program: urban food gardening is clearly something residents are passionate about. On the other hand, the existence and popularity of Brampton’s Backyard Garden Program reflect municipal priorities: food gardening is found throughout the city’s policy documents, and there are other municipally sponsored food gardening programs running at City Hall and fire stations (Feinstein, 2021; Partners in Project Green, 2015). Brampton’s community garden program is one of the best in the country in that the city runs and finances gardens, but also supplies land, materials, and city staff labor to help organizations set up new gardens (City of Brampton, 2021c).

### Municipal home food gardening in 2021

Brampton and Victoria ran their home food gardening programs again in 2021, and Halifax replaced its program with a new food gardening initiative. Encouragingly, Brampton has already decided to run its programs again in 2022. The city has gone a step further by making the Backyard Garden Program an official line item in its operating budget until at least 2024 (which is all the budget forecasting completed to this point) (Whillans, pers. comm., 2021).

Brampton modified its programs in 2021 to consider lessons learned from 2020. It capped participation at 3000 households and residents all received the same gardening kit, which contained 140 l of bagged soil and 3 packages of vegetable seeds. Registration for the Backyard Garden program opened 1 February 2021, and all kits were claimed within 31 h; participants from 2020 were not allowed to receive free seeds and soil (City of Brampton, 2021b). Get Growing, Victoria! is essentially unmodified: 10 seedling distribution days took place in June, with more to come in late August and early September (City of Victoria, 2021a).

Halifax’s gardening initiative for 2021 was not focused on food security, but, rather, on neighborhood social cohesion. The municipality’s long-running Neighborhood Placemaking program provides material and funding support for neighborhood gathering places and activities, such as street painting and building outdoor movie screens. In 2021, since the usual Placemaking projects were on hold because of the pandemic, Halifax offered gardening kits containing thousands of vegetable seeds to share among households on a single street or neighborhood-wide, as well as virtual training sessions with staff horticulturalists. There are no restrictions on participation, except that Haligonians must apply with at least one neighbor household (Halifax Regional Municipality, 2021b).

The municipalities that did not run their home food gardening program in 2021 (including Halifax) cited the same reason: funding. When the financial situation of municipalities is considered on top of pandemic conditions (Hachard, 2020; Tolley and Young, 1991), the speed with which these 19 municipalities developed and implemented their gardening programs is even more impressive. Under provincial law, municipalities are not permitted to run a deficit, so in 2020 and 2021, this required Herculean efforts on the part of municipal finance staff. The COVID-19 pandemic forced the creation of entirely new necessary services or forced existing services to change in significant ways. The federal and provincial/territorial governments allocated millions of dollars to municipalities to help mitigate the economic fallout, reduce operating deficits, and enable municipalities to construct meaningful budgets for 2021 and forecasts for the following two to three fiscal years. However, the federal-provincial/territorial Safe Restart Agreement was announced at the end of July 2020, with exact amounts for specific municipalities worked out and transferred throughout the Fall (Intergovernmental Affairs Canada, 2020; Municipal Affairs and Housing Nova Scotia, 2020; Municipal Affairs and Housing Ontario, 2021; Union of BC Municipalities, 2020). In July 2020, municipalities were already three and a half months into their response to the pandemic, and every one of the municipal home food gardening programs was in progress or already completed (from the point of view of the municipality—home gardeners were, of course, tending their crops).

Owing to their subservient relationship to provincial and territorial governments, municipalities are limited in their ability to earn money to pay for operating costs and all the services they provide to residents. In 2020, Halifax navigated these restrictions by using funds and staff from community engagement programs that had to be canceled because of the pandemic; gardening kit supplies cost approximately $3500. Municipal staff hired under the Youth Live scheme were tasked with assembling the gardening kits and assisting community partner organizations with distribution because their existing jobs were also canceled. Halifax also received a bulk purchase discount on vegetable seeds and bags of soil (HRM staff member, pers. comm., 2021).

The total cost for a home food gardening program is difficult to ascertain because of the in-kind factor—so many municipal staff members were redirected from their usual tasks to support the food gardening initiative. Of course, many municipalities partnered with non-profit organizations, which adds another layer to cost. Montréal knew the exact direct cash amounts—$45,000—but that does not include any staff time. Halifax’s cost calculation does not include the hours of work other municipal staff contributed, nor does it factor in delivery of kits to community distribution partners. Similarly, the cost of Get Growing, Victoria!, did not make data on costs publicly available, making cost-estimates difficult given the number of labor hours: growing over 81,000 healthy vegetable seedlings and preparing hundreds of yards of soil is not a quick project. Whillans estimates that the upfront cost for Brampton’s Backyard Garden Program was around $100,000, but that does not include the in-kind contributions of city staff, which amounted to hundreds of hours of labor. At least six city departments were involved, with dozens of staff members (City of Brampton, 2021b; Whillans, pers. comm., 2021). In 2021, Brampton’s Backyard Garden Program was sponsored by several businesses, which greatly reduced the upfront cost of the program (City of Brampton, 2021a).

### Limitations

This study has limitations. Municipalities with populations <2000 were not included. The size was determined by the 2016 Statistics Canada census data. Smaller municipalities’ resources are limited, due to the small tax base (Gadenne, 2017; Knox and Mayer, 2013; Martinez-Fernandez et al., 2012) resulting in reduced programming and staff. In addition, smaller municipalities in Canada tend to be rural, situated in farmland or in northern regions (Beckie et al., 2012). While rural food security is a concern, it represents different challenges to urban food security including access to transportation, employment opportunities, and income support (Andrée et al., 2016; Garasky et al., 2004). Further research on residential food production in rural settings would be needed to identify specific challenges faced by small Canadian municipalities.

## Conclusion

Municipal home food gardening programs in 2020 arose in response to a specific set of circumstances, namely, a global pandemic and related concerns about food systems and an increase in food insecurity. This study shows that enabling and supporting home food gardening was both an appropriate and advantageous response to the pandemic and, moreover, that municipalities should have home food gardening programs every year. The 19 programs examined here vary widely in their scope and methods and are only partially aligned on their stated aims; some programs were more successful than others, both in terms of participant and facilitator satisfaction and in the number of participants reached. All these programs reveal the potential for municipalities to make immediate and obviously positive contributions to the lives of their citizens, with a relatively low price tag. Each of these 19 programs struggled with inclusivity and representation of diverse population groups, but those faults could be easily fixed. Overall, municipal home food gardening programs aid household, neighborhood, and community food security. Home food gardening programs also inspire familial and neighborhood togetherness, foster civic pride, and create connections between local government, non-profit organizations, and residents. Gardening also promotes sustainable living practices, benefiting environmental protection, conservation, and improvement. Home food gardening initiatives meet policy goals in several broad areas, including the health and wellness of communities, environmental sustainability, strengthening local food systems, and progressive land use. As these 19 municipal home food gardening programs demonstrate, home food gardening can have a positive impact on the citizens of a municipality in meaningful ways—global crisis or not.

## Data Availability

The datasets generated during the current study are not publicly available to protects participants’ privacy and due to the sensitive nature of the issues discussed. The anonymized excerpts on which the present analysis is based are included in the text of this paper.

## References

[CR1] Alaimo K, Beavers AW, Crawford C, Snyder EH, Litt JS (2016). Amplifying health through community gardens: a framework for advancing multicomponent, behaviorally based neighborhood interventions. Curr Environ Health Rep.

[CR2] Al-Delaimy WK, Webb M (2017). Community gardens as environmental health interventions: benefits versus potential risks. Curr Environ Health Rep.

[CR3] Algert SJ, Baameur A, Diekmann LO, Gray L, Ortiz D (2016). Vegetable output, cost savings, and nutritional value of low-income families’ home gardens in San Jose, CA. J Hunger Environ Nutr.

[CR4] Andrée P, Langille L, Clement C, Williams P, Norgang E (2016). Structural constraints and enablers to community food security in Nova Scotia, Canada. J Hunger Environ Nutr.

[CR5] Beckie MA, Kennedy EH, Wittman H (2012). Scaling up alternative food networks: farmers’ markets and the role of clustering in western Canada. Agric Hum Values.

[CR6] Brehaut L (2020) Boom in pandemic gardening has led to shortage of mason jars and lids. National Post. https://nationalpost.com/life/food/boom-in-pandemic-gardening-has-led-to-shortage-of-mason-jars-and-lids

[CR7] Brown B (2020) Nunavut greenhouse could teach scientists how to grow food in outer space | CBC News. CBC. https://www.cbc.ca/news/canada/north/space-agency-teaches-gjoa-haven-growing-food-in-harsh-climates-1.5681294

[CR8] Cabannes Y, Marocchino C (eds.) (2018) Integrating food into urban planning. UCL Press and Food and Agriculture Organization of the United Nations

[CR9] CBC News (2021) Seed shortages return as pandemic gardeners get their hands dirty. CBC. https://www.cbc.ca/news/canada/calgary/calgary-seed-shortages-pandemic-1.5955821

[CR10] Chalmin-Pui LS, Roe J, Griffiths A, Smyth N, Heaton T, Clayden A, Cameron R (2021). “It made me feel brighter in myself”—the health and well-being impacts of a residential front garden horticultural intervention. Landsc Urban Plan.

[CR11] City of Brampton (2020) Brampton Grow Green: Environmental Master Plan. https://www.brampton.ca/EN/residents/GrowGreen/Documents/EMP/Brampton_Grow_Green_EMP_2020_Final.pdf

[CR12] City of Brampton (2021a) Backyard Garden Program. https://www.brampton.ca/EN/residents/parks/Pages/Backyard-Gardens.aspx#ad-image-0

[CR13] City of Brampton (2021b) Community Backyard Garden Program Manual

[CR14] City of Brampton (2021c) Community Gardens Information Handbook. https://www.brampton.ca/EN/residents/parks/Documents/Community%20Gardens/Community-Garden-Handbook.pdf

[CR15] City of North Battleford (2020) Agenda for Council Meeting. https://www.cityofnb.ca/mrws/filedriver/Agendas_and_Agenda_Sheets/C82-2020.pdf

[CR16] City of Victoria (2021a) Get Growing, Victoria! https://www.victoria.ca/EN/main/residents/parks/growing-in-the-city/get-growing-victoria.html

[CR17] City of Victoria (2021b) Urban Food Table. https://www.victoria.ca/EN/main/city/other-boards-committees/urban-food-table.html

[CR18] Cochran S, Minaker L (2020). The value in community gardens: a return on investment analysis. Can Food Stud/La Re Can Étud Sur l’alimentation.

[CR19] CoDyre M, Fraser EDG, Landman K (2015). How does your garden grow? An empirical evaluation of the costs and potential of urban gardening. Urban For Urban Green.

[CR20] Cultiver Montréal (2020) Bilan de Mi-Saison 2020. https://www.cultivermontreal.ca/

[CR21] Cultiver Montréal (2021) Homepage. https://www.cultivermontreal.ca/

[CR22] Duchemin E (2020) 43% des familles québécoises ont un potager [Billet]. AgriUrbain. https://agriurbain.hypotheses.org/4864

[CR23] Duchemin E, McClintock N (2020) L’apport alimentaire de l’agriculture urbaine sociale des villes en temps de crise: Le cas de Montréal. AgriUrbain. https://agriurbain.hypotheses.org/4739

[CR24] Feinstein C (2021) Brampton approves first Off-Grid Organic Food Shed. Thestar.Com. https://www.thestar.com/local-brampton/news/2021/05/08/brampton-approves-first-off-grid-organic-food-shed.html

[CR25] FoodARC (2021) Dismantling stigma. https://foodarc.ca/projects/current-projects/dismantling-stigma

[CR26] Gadenne L (2017). Tax me, but spend wisely? Sources of public finance and government accountability. Am Econ J.

[CR27] Garasky S, Morton LW, Greder K (2004). The food environment and food insecurity: perceptions of rural, suburban, and urban food pantry clients in Iowa. Fam Econ Nutr Rev.

[CR28] Garcia MT, Ribeiro SM, Germani ACCG, Bógus CM (2018). The impact of urban gardens on adequate and healthy food: a systematic review. Public Health Nutr.

[CR29] Genter C, Roberts A, Richardson J, Sheaff M (2015). The contribution of allotment gardening to health and wellbeing: a systematic review of the literature. Br J Occup Ther.

[CR30] Godrich SL, Loewen OK, Blanchet R, Willows N, Veugelers P (2019). Canadian children from food insecure households experience low self-esteem and self-efficacy for healthy lifestyle choices. Nutrients.

[CR31] Gray L, Guzman P, Glowa KM, Drevno AG (2014). Can home gardens scale up into movements for social change? The role of home gardens in providing food security and community change in San Jose, California. Local Environ.

[CR32] Gregory MM, Leslie TW, Drinkwater LE (2016). Agroecological and social characteristics of New York City community gardens: contributions to urban food security, ecosystem services, and environmental education. Urban Ecosyst.

[CR33] Hachard T (2020) Opinion: pandemic pressures reveal cracks in Canadian federalism around municipal government powers, responsibilities|CBC News. CBC. https://www.cbc.ca/news/opinion/opinion-federal-provincial-municipal-powers-1.5813461

[CR34] Halifax Regional Municipality (2019) Community Mobilization Teams. https://www.halifax.ca/sites/default/files/documents/about-the-city/regional-community-planning/_CMT_Poster_18x24_Final_0.pdf

[CR35] Halifax Regional Municipality (2021a) Working together to promote community food security. https://www.halifax.ca/about-halifax/regional-community-planning/halifax-food-action

[CR36] Halifax Regional Municipality (2021b) Neighbourhood placemaking. https://www.halifax.ca/parks-recreation/arts-culture-heritage/community-arts/neighbourhood-placemaking

[CR37] Hartwig KA, Mason M (2016). Community gardens for refugee and immigrant communities as a means of health promotion. J Commun Health.

[CR38] Helmer J (2020) How the coronavirus pandemic has led to a boom in crisis gardening. HuffPost. https://www.huffpost.com/entry/seeds-crisis-gardening-coronavirus-food_n_5e85eca0c5b6f55ebf492212

[CR39] Intergovernmental Affairs Canada (2020) The safe restart agreement. https://www.canada.ca/en/intergovernmental-affairs/services/safe-restart-agreement.html

[CR40] Kingsley J, Foenander E, Bailey A (2020). “It’s about community”: exploring social capital in community gardens across Melbourne, Australia. Urban For Urban Green.

[CR41] Klinkenberg M (2020) Seed companies thought coronavirus would ruin their businesses. Instead, demand has soared. Globe Mail. https://www.theglobeandmail.com/life/article-peis-veseys-sees-demand-soar-as-covid-19-spurs-vegetable-seeds-frenzy/

[CR42] Knox P, Mayer H (2013) Small Town Sustainability: 2nd, revised and enlarged edition Birkhäuser. 10.1515/9783038210283

[CR43] Kortright R, Wakefield S (2011). Edible backyards: a qualitative study of household food growing and its contributions to food security. Agric Hum Values.

[CR44] Kroeker A (2017) Finding their new land: site selection and conceptual design for an urban farm for newcomers in Winnipeg’s inner city. https://mspace.lib.umanitoba.ca/xmlui/handle/1993/32665

[CR45] Lai J, Widmar NO (2021). Revisiting the digital divide in the COVID-19 era. Appl Econ Perspect Policy.

[CR46] Lal R (2020). Home gardening and urban agriculture for advancing food and nutritional security in response to the COVID-19 pandemic. Food Security.

[CR47] Lowan-Trudeau M, Keough N, Wong J, Haidey S (2020). The affordable housing, transportation, and food nexus: community gardens and healthy affordable living in Calgary. Can Geogr/Le Géographe Canadien.

[CR48] Lucas L, Li F (2020). Growing food, sharing culture at the Rainbow Community Garden in Winnipeg, Canada. Can Food Stud/La Rev Canadienne Des Études Sur l’alimentation.

[CR49] Martinez-Fernandez C, Audirac I, Fol S, Cunningham-Sabot E (2012). Shrinking cities: urban challenges of globalization. Int J Urban Region Res.

[CR50] Mullins L, Charlebois S, Finch E, Music J (2021). Home food gardening in Canada in response to the COVID-19 pandemic. Sustainability.

[CR51] Municipal Affairs and Housing Nova Scotia (2020) COVID-19 support for municipalities. News Releases. https://novascotia.ca/news/release/?id=20201104002

[CR52] Municipal Affairs and Housing Ontario (2021) Ontario provides additional support for municipalities during COVID-19. News.Ontario.Ca. https://news.ontario.ca/en/release/60538/ontario-provides-additional-support-for-municipalities-during-covid-19

[CR53] Music J, Finch E, Gone P, Toze S, Charlebois S, Mullins L (2021). Pandemic victory gardens: potential for local land use policies. Land Use Policy.

[CR54] Olson I (2020) Montreal to help residents grow their own food this summer to inspire self-sufficiency. CBC https://www.cbc.ca/news/canada/montreal/community-gardens-montreal-food-production-1.5551546

[CR55] Partners in Project Green (2015) Up on the roof: bringing urban agriculture to Brampton City Hall. Partners in Project Green https://partnersinprojectgreen.com/news/up-on-the-roof-bringing-urban-agriculture-to-brampton-city-hall/

[CR56] Pigalak R (2020) Mini greenhouses popping up in Cambridge Bay. NUNAVUT NEWS. https://www.nunavutnews.com/nunavut-news/mini-greenhouses-popping-up-in-cambridge-bay/

[CR57] PROOF (2019) Relationship between food banks and food insecurity in Canada. https://proof.utoronto.ca/wp-content/uploads/2019/11/PROOF_FACTSHEET_Foodbanks-112019.pdf

[CR58] Radio-Canada (2021) La folie des semences est lancée en Estrie. Radio-Canada.ca. https://ici.radio-canada.ca/nouvelle/1765442/jardin-semence-gaillarde-estrie-serre-saint-elie

[CR59] Reddick CG, Enriquez R, Harris RJ, Sharma B (2020). Determinants of broadband access and affordability: an analysis of a community survey on the digital divide. Cities.

[CR60] Reese AM (2018). “We will not perish; we’re going to keep flourishing”: race, food access, and geographies of self-reliance. Antipode.

[CR61] Rosan CD, Pearsall H (2017) Growing a sustainable city? The question of urban agriculture. University of Toronto Press

[CR62] Sharpe K (2021) Retailers stocking up ahead of a busy gardening season. CTV News. https://kitchener.ctvnews.ca/retailers-stocking-up-ahead-of-a-busy-gardening-season-1.5360641

[CR63] Smith JP, Meerow S, Turner BL (2021). Planning urban community gardens strategically through multicriteria decision analysis. Urban For Urban Green.

[CR64] Smith VM, Greene RB, Silbernagel J (2013). The social and spatial dynamics of community food production: a landscape approach to policy and program development. Landsc Ecol.

[CR65] Soderholm D (2015) Planning for urban agriculture in Canadian cities. Thesis, University of Waterloo

[CR67] Statistics Canada (2019) Census Profile, 2016 Census. https://www12.statcan.gc.ca/census-recensement/2016/dp-pd/prof/index.cfm?Lang=E

[CR68] Taylor JR, Lovell ST (2014). Urban home food gardens in the Global North: research traditions and future directions. Agric Hum Values.

[CR69] Taylor JR, Lovell ST (2015). Urban home gardens in the Global North: a mixed methods study of ethnic and migrant home gardens in Chicago, IL. Renew Agric Food Syst.

[CR70] Tharrey M, Sachs A, Perignon M, Simon C, Mejean C, Litt J, Darmon N (2020). Improving lifestyles sustainability through community gardening: results and lessons learnt from the JArDinS quasi-experimental study. BMC Public Health.

[CR71] Theodorou A, Panno A, Carrus G, Carbone GA, Massullo C, Imperatori C (2021). Stay home, stay safe, stay green: the role of gardening activities on mental health during the Covid-19 home confinement. Urban For Urban Green.

[CR72] Tiku N (2021). A world without digital inclusivity: What it means to securitize technology. J Intell Confl Warfare.

[CR73] Tolley E, Young WR (1991) Municipalities, the constitution, and the Canadian federal system [Background Paper]. Library of Parliament. https://publications.gc.ca/Collection-R/LoPBdP/BP-e/bp276-1e.pdf

[CR74] Toughill K (2018) Putting down roots: how community gardens help immigrant retention. Public Policy Forum. https://ppforum.ca/articles/putting-down-roots-how-community-gardens-help-immigrant-retention/

[CR75] Union of BC Municipalities (2020) Safe-Restart-funds-for-local-government-operations-distributed. https://www.ubcm.ca/EN/meta/news/news-archive/2020-archive/safe-restart-funds-for-local-government-operations-distributed.html

[CR76] Ville de Montréal (2020) Opening of community gardens: the city announces various measures to improve food security [Web page]. Ville de Montréal. http://ville.montreal.qc.ca/portal/page?_pageid=5977,43117560&_dad=portal&_schema=PORTAL&id=32606

[CR77] Winnipeg Food Council (2020) Meadowood Victory Garden Report. https://winnipeg.ca/clerks/boards/WpgFoodCouncil/pdfs/MeadowoodVictoryGardenReport.pdf

